# Exploration of the underlying biological differences and targets in ovarian cancer patients with diverse immunotherapy response

**DOI:** 10.3389/fimmu.2022.1007326

**Published:** 2022-09-15

**Authors:** Jinjin Chen, Surong Chen, Xichao Dai, Liang Ma, Yu Chen, Weigang Bian, Yunhao Sun

**Affiliations:** ^1^ Oncology Department, The First People’s Hospital of Yancheng City, The Yancheng Clinical College of Xuzhou Medical University, Yancheng, China; ^2^ Department of Thoracic Surgery, The First People’s Hospital of Yancheng City, The Yancheng Clinical College of Xuzhou Medical University, Yancheng, China

**Keywords:** ovarian cancer, immunotherapy response, cancer-associated fibroblasts, machine learning, prognosis

## Abstract

**Background:**

Preclinical trials of immunotherapy in ovarian cancer (OC) have shown promising results. This makes it meaningful to prospectively examine the biological mechanisms explaining the differences in response performances to immunotherapy among OC patients.

**Methods:**

Open-accessed data was obtained from the Cancer Genome Atlas and Gene Expression Omnibus database. All the analysis was conducted using the R software.

**Results:**

We firstly performed the TIDE analysis to evaluate the immunotherapy response rate of OC patients. The machine learning algorithm LASSO logistic regression and SVM-RFE were used to identify the characteristic genes. The genes DPT, RUNX1T1, PTPRN, LSAMP, FDCSP and COL6A6 were selected for molecular typing. Our result showed that the patients in Cluster1 might have a better prognosis and might be more sensitive to immunotherapy, including PD-1 and CTLA4 therapy options. Pathway enrichment analysis showed that in Cluster2, the pathway of EMT, TNFα/NF-kB signaling, IL2/STAT5 signaling, inflammatory response, KRAS signaling, apical junction, complement, interferon-gamma response and allograft rejection were significantly activated. Also, genomic instability analysis was performed to identify the underlying genomic difference between the different Cluster patients. Single-cell analysis showed that the DPT, COL6A6, LSAMP and RUNX1T1 were mainly expressed in the fibroblasts. We then quantified the CAFs infiltration in the OC samples. The result showed that patients with low CAFs infiltration might have a lower TIDE score and a higher proportion of immunotherapy responders. Also, we found all the characteristic genes DPT, RUNX1T1, PTPRN, LSAMP, FDCSP and COL6A6 were upregulated in the patients with high CAFs infiltration. Immune infiltration analysis showed that the patients in Cluster2 might have a higher infiltration of naive B cells, activated NK cells and resting Dendritic cells.

**Conclusions:**

In summary, our study provides new insights into ovarian cancer immunotherapy. Meanwhile, specific targets DPT, RUNX1T1, PTPRN, LSAMP, FDCSP, COL6A6 and CAFs were identified for OC immunotherapy.

## Introduction

Ovarian cancer (OC) represents the seventh most frequent women malignancies around the world (1). Multiple factors contribute to the development of OC, including hormone levels, reproductive factors, genetic susceptibility, environmental exposure, and lifestyle (1). For earl-stage OC, surgery remains the best treatment option and can improve patient long-term survival (2). However, only about 20% of OV patients can be diagnosed and treated early due to unusual symptoms (2). Unfortunately, due to the characteristics of high invasion and metastasis, the prognosis of advanced OC is extremely poor (3).

Combined palliative surgery and chemotherapy are often used to treat advanced OC, aiming to reduce patient pain and prolong survival. In many cases, however, this benefit is limited (4). Despite the use of targeted therapy drugs such as bevacizumab and PARP inhibitors in OC treatment, the 5-year survival rate is still less than 50% (5). Moreover, over the past few decades, survival rates for OC have not been significantly increased (5). There has been considerable progress in immunotherapy in the past ten years, bringing revolutionary changes to the management of solid tumors (6). Although immunotherapy for OC has not been approved yet, with the rapid development of immune checkpoint blockade, cancer vaccine and adoptive cell therapy, there have been a large number of pre-clinical trials of OC immune checkpoint inhibitor therapy, for example, NCT03353831, NCT01772004 and others (7). According to tumor biomarker stratification, identifying sensitive/resistant subgroups might improve immunotherapy response prediction. In light of the experience of other solid tumors and preclinical trials of immunotherapy for OC, these markers mainly include tumor mutation load, PD-L1, tumor infiltrating lymphocytes, homologous recombination defects, and intratumor neoantigen heterogeneity (8). Using these biomarkers to select ideal immunotherapy candidates may be the future of OC treatment.

Researchers have great convenience to investigate further with the rapid development of bioinformatics technology (9). In our study, we performed the TIDE analysis to evaluate the immunotherapy response rate of OC patients. The machine learning algorithm LASSO logistic regression and SVM-RFE were used to identify the characteristic genes. The genes DPT, RUNX1T1, PTPRN, LSAMP, FDCSP and COL6A6 were selected for molecular typing. Our result showed that the patients in Cluster1 might have a better prognosis and might be more sensitive to immunotherapy, including PD-1 and CTLA4 therapy options. Pathway enrichment analysis and genomic instability analysis were performed to identify the underlying biological difference between the different Cluster patients. Single-cell analysis showed that the DPT, COL6A6, LSAMP and RUNX1T1 were mainly expressed in the fibroblasts. Next, we found that the patients with low CAFs infiltration might have a lower TIDE score and a higher proportion of immunotherapy responders.

## Methods

### Data assessment

A comprehensive retrieval and data quality evaluation of the public database was carried out when the study began. Data quality assessment includes i). Probe numbers; ii). Expression profile magnitude; iii) Clinical information. Finally, the open-accessed data of The Cancer Genome Atlas (TCGA), as well as GSE51088 (10) and GSE53963 (11) from the Gene Expression Omnibus (GEO) database were selected. Detailed, the transcriptional profiling data were “STAR-Counts” form and the clinical information was “bcr-xml” form. The expression profile of GSE51088 and GSE53963 were downloaded from the link of “Series Matrix File(s)” and annotated based on the platform files (GSE51088: GPL7264; GSE53963: GPL6480). Sva package was utilized for data combination and batch effect reduction. The basic information of the enrolled patients was shown in **Table 1**.

**Table 1:** Basic information of enrolled patients.

**Clinical Features**	**Number of patients (n)**	**Percentage (%)**
Age		
<=60	326	55.5%
>60	261	44.5%
Grade		
G1	6	1.0%
G2	69	11.8%
G3	495	84.3%
G4	1	0.2%
Unknown	16	2.7^

### Tumor immune dysfunction and exclusion

TIDE algorithm was performed to predict the underlying immunotherapy response of OV patients (http://tide.dfci.harvard.edu/). All the patients were assigned a TIDE score, in which TIDE > 0 were defined as immunotherapy non-responder and < 0 were defined as immunotherapy responders (12, 13). The evaluation of the patient’s response to PD-1 and CTLA4 therapy was conducted through submap analysis, which is an unsupervised subclass mapping method that reveals common subtypes between independent datasets (https://cloud.genepattern.org/gp).

### Machine learning algorithm and molecular subtyping

The machine learning algorithms, including LASSO logistic regression and support vector machine recursive feature elimination (SVM-RFE) were used to identify the characteristic genes (14, 15). Molecular subtyping was conducted based on the ConsensusClusterPlus package in R software.

### Pathway enrichment analysis and genomic instability

Gene Set Enrichment Analysis (GSEA) was performed to compare the underlying biological differences between the two groups (16). The reference gene set was Hallmark, c2.cp.kegg.v7.5.1.symbols and c5.go.v7.5.1.symbols gene sets obtained from https://www.gsea-msigdb.org/gsea/downloads.jsp. Genomic instability analysis was evaluated, including the tumor mutation burden (TMB), microsatellite instability (MSI) and tumor stemness (mRNAsi and EREF-mRNAsi). ClueGO analysis is a plug-in of Cytoscape that could decipher functionally grouped gene ontology and pathway annotation networks (17).

### Single sample gene set enrichment and immune infiltration analysis

Single sample gene set enrichment analysis (ssGSEA) was used to quantify the infiltration of cancer-associated fibroblasts (CAFs) (18). The reference genes was shown in **Supplementary Table S1**. CIBERSORT algorithm was used to quantify 22 immune cell infiltration of OC immune microenvironment (19).

### Single-cell level

The analysis of the characteristic genes at the single cell level was based on the Tumor Immune Single-cell Hub website (TISCH, http://tisch.comp-genomics.org/). With TISCH, cell-type annotations at the single-cell level are available, allowing exploration of tumor microenvironments (TME) across a variety of cancer types.

### Statistical analysis

All the statistical analysis was performed in R software. Kaplan-Meier (KM) survival curve was used to compare the prognosis difference between two groups. The receiver operating characteristic (ROC) curve was utilized to evaluate the prediction ability of specific features. The significance of a difference was determined by the p-value (p < 0.05). Student T-tests were performed on data with normal distribution. Non-normal distributions were tested with the Mann-Whitney U test.

## Results

### Identification of the characteristic gene of immunotherapy response

**Figure 1:**
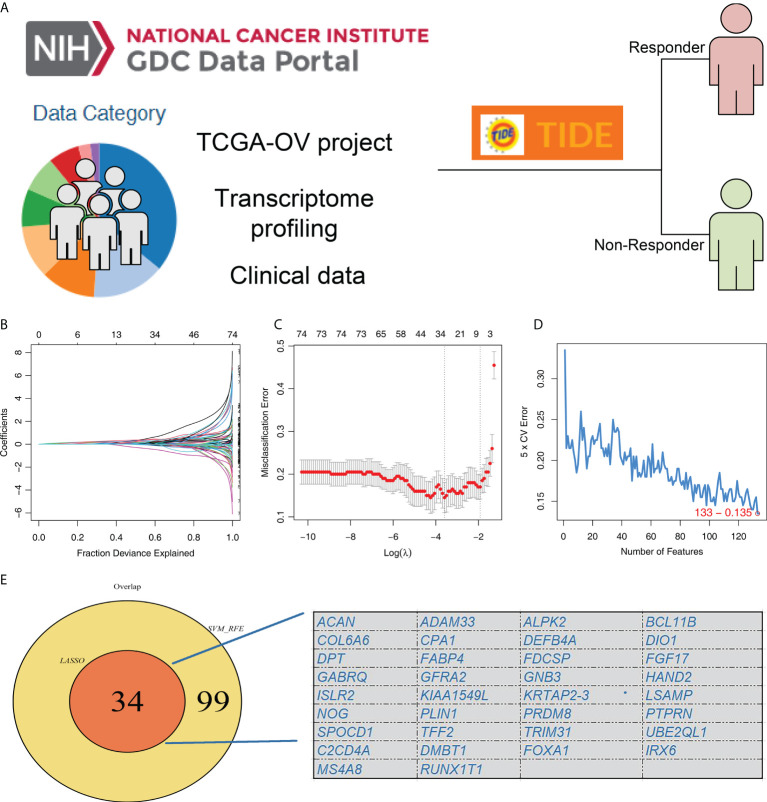
Identification of the characteristic gene of immunotherapy response. **(A)** TIDE analysis was performed to evaluate the immunotherapy response of TCGA-OC patients, in which TIDE > 0 were defined as immunotherapy non-responder and < 0 were defined as immunotherapy responders; **(B, C)** LASSO logistic regression algorithm; **(D)** SVM-RFE algorithm; **(E)** LASSO logistic regression and SVM-RFE algorithms identified 34 characteristic genes.

The flow chart of our whole study was shown in **Supplementary Figure S1**. TIDE analysis was firstly performed based on the OC patients in TCGA database, in which TIDE > 0 were defined as immunotherapy non-responder and < 0 were defined as immunotherapy responders (**Figure 1A**). LASSO logistic regression and SVM-RFE algorithms were utilized to screen the characteristic genes of patients in the immunotherapy responder group (**Figures 1B-D**). Finally, these two algorithms identified 34 characteristic genes (**Figure 1E**).

### Molecular typing

**Figure 2:**
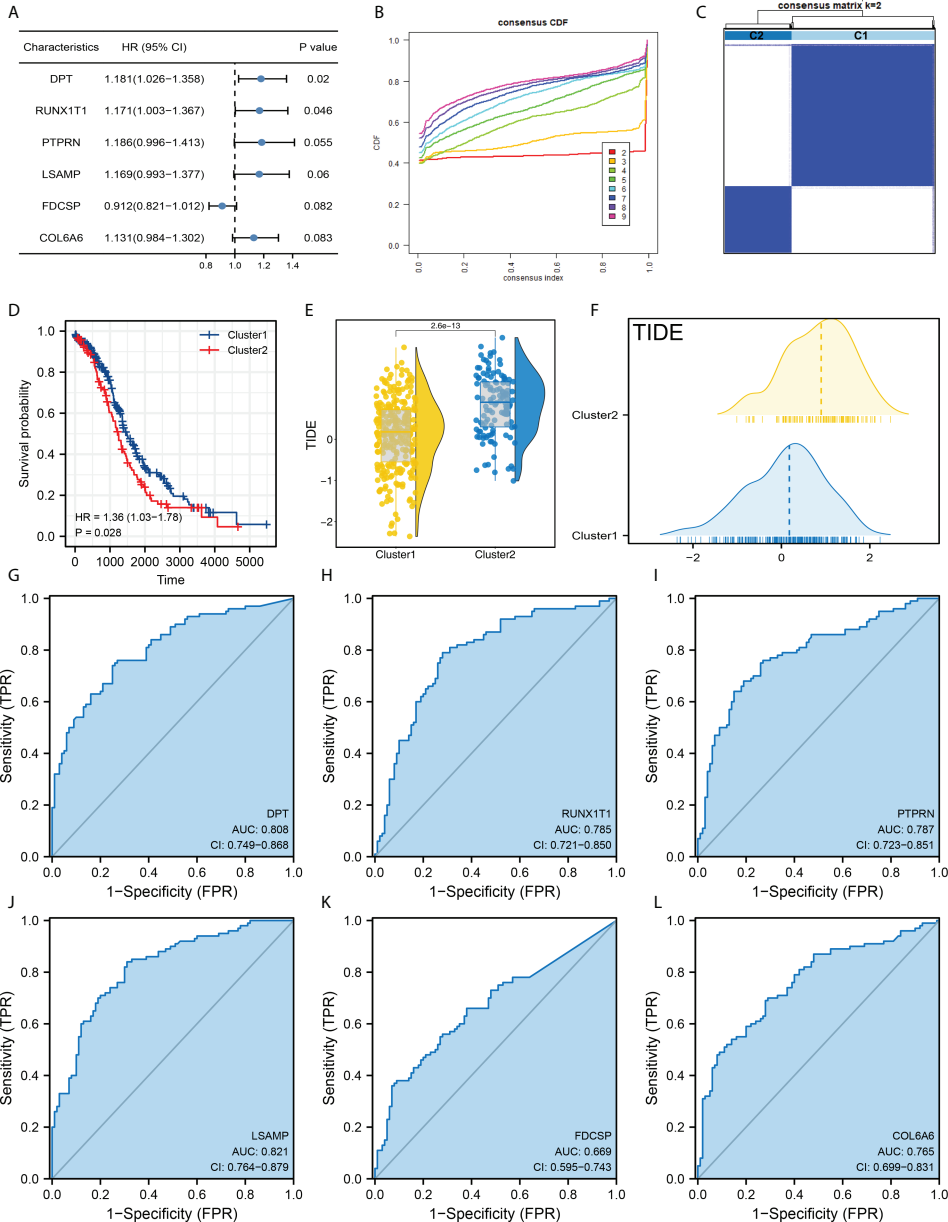
Molecular typing based on DPT, RUNX1T1, PTPRN, LSAMP, FDCSP and COL6A6. **(A)** Among all the characteristic genes, DPT, RUNX1T1, PTPRN, LSAMP, FDCSP and COL6A6 were identified for their prognosis correlation (P < 0.05); **(B)** ConsensusClusterPlus package was used for molecular typing in the patients of TCGA database; **(C)** Dividing patients into two subtypes provides the best differentiation; **(D)** KM survival curve of patients in Cluster1 and Cluster2; **(E, F)** The patients in Cluster2 had a higher TIDE score than Cluster1; **(G–L)** The prediction ability of DPT, RUNX1T1, PTPRN, LSAMP, FDCSP and COL6A6 on patients immunotherapy response.

Our goal is to identify the patients with different prognosis and immunotherapy response rates by clustering samples. Next, we performed the univariate Cox regression analysis and the characteristic genes DPT, RUNX1T1, PTPRN, LSAMP, FDCSP and COL6A6 were identified for molecular typing (**Figure 2A**). In detail, the ConsensusClusterPlus package was used for molecular typing in the patients of TCGA database (**Figure 2B** and **Supplementary Figure S2**). In all subtypes, dividing patients into two subtypes provides the best differentiation (**Figure 2C**). The KM survival curve showed that the patients in Cluster2 might have a worse prognosis (**Figure 2D**). Also, we found that the patients in Cluster2 might have a higher TIDE score than those in Cluster1 (**Figures 2E, F**). Moreover, DPT, RUNX1T1, PTPRN, LSAMP, FDCSP and COL6A6 all showed a good prediction ability of patients immunotherapy response (**Figures 2G-L**, DPT, AUC = 0.808; RUNX1T1, AUC = 0.785; PTPRN, AUC = 0.787; LSAMP, AUC = 0.821; FDCSP, AUC = 0.669; COL6A6, AUC = 0.765).

### Patients in Cluster1 are more sensitive to immunotherapy

**Figure 3:**
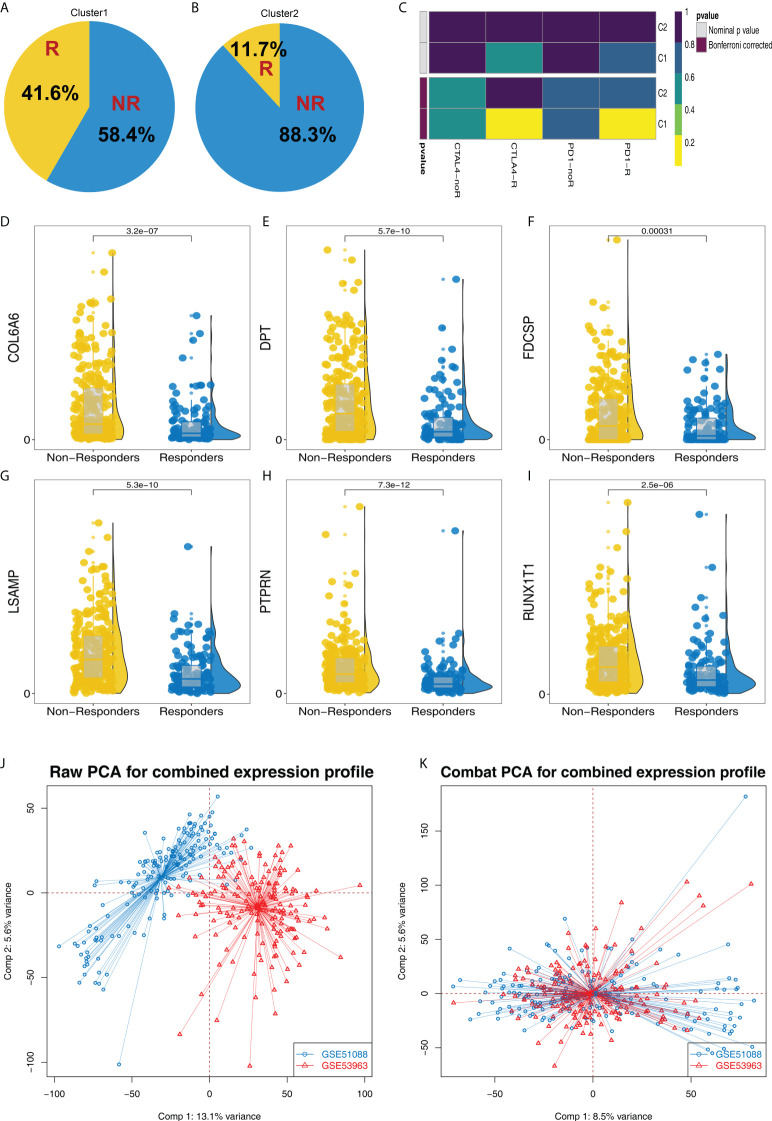
Cluster1 and Cluster2 had different immunotherapy response. **(A, B)** The proportion of immunotherapy responders and non-responders in Cluster1 and Cluster2 patients; **(C)** Submap algorithm indicated that the Cluster1 patients are sensitive to both PD-1 and CTLA4 therapy; **(D–I)** The expression level of DPT, RUNX1T1, PTPRN, LSAMP, FDCSP and COL6A6 in immunotherapy responders and non-responders; **(J–K)** Sva package was used for data combination and batch effect reduction of GSE51088 and GSE53963.

According to the TIDE result, we found that the proportion of immunotherapy responders in Cluster1 is 41.6%, which is greatly higher than the 11.7% in Cluster2 (**Figures 3A, B**). Submap algorithm indicated that the Cluster1 patients are sensitive to both PD-1 and CTLA4 therapy (**Figure 3C**). Meanwhile, DPT, RUNX1T1, PTPRN, LSAMP, FDCSP and COL6A6 all showed a higher expression level in immunotherapy non-responders patients (**Figures 3D-I**). Furthermore, we try to validate our results in the GSE cohorts. GSE51088 and GSE53963 were selected (**Figure 3J**). Sva package was used for data combination and batch effect reduction (**Figure 3K**).

### Validation in combined GSE cohorts

**Figure 4:**
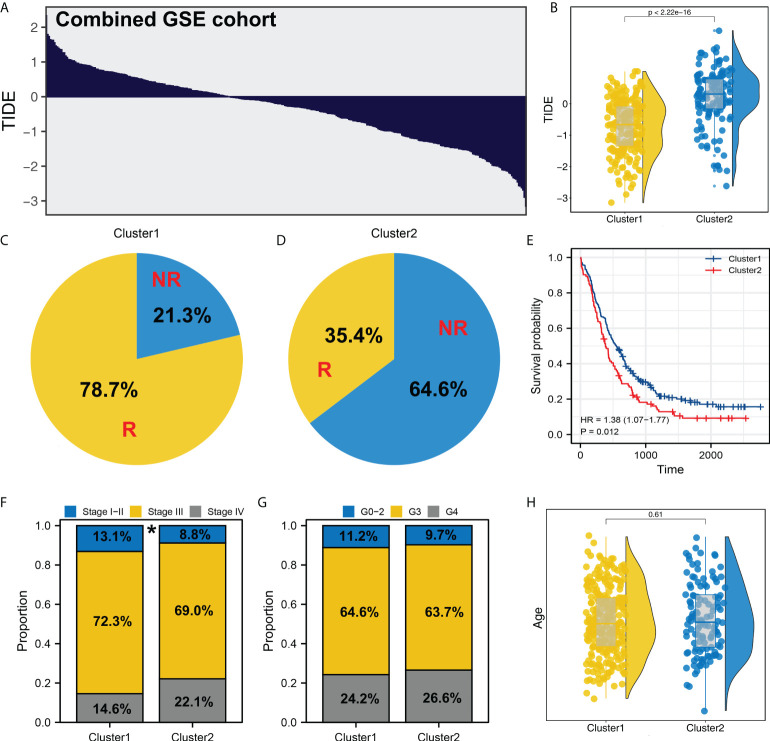
Validation in GSE cohort. **(A)** TIDE analysis was performed in the combined GSE cohort; **(B)** In the GEO cohort, Cluster2 also had a higher TIDE score; **(C, D)** The proportion of immunotherapy responders and non-responders in Cluster1 and Cluster2 patients; **(E)** KM survival curve of Cluster1 and Cluster2 patients; **(F–H)** Clinical differences between Cluster1 and Cluster2 (gender, age and grade). * = P < 0.05.

We next performed the TIDE analysis in the combined GSE cohort (**Figure 4A**). Same with the result in TCGA, the patients in Cluster1 had a lower TIDE score and a higher proportion of immunotherapy responders than those in Cluster2 (**Figures 4B-D** and **Supplementary Figure S3**). KM survival curve showed that the patients in Cluster2 might have a worse survival (**Figure 4E**). Meanwhile, clinical correlation analysis showed that the patients in Cluster2 might have a more progressive clinical stage, but not pathological grade (**Figures 4F, G**). Meanwhile, no significant difference was observed between the patients in different age group (**Figure 4H**).

### Pathway enrichment analysis

**Figure 5:**
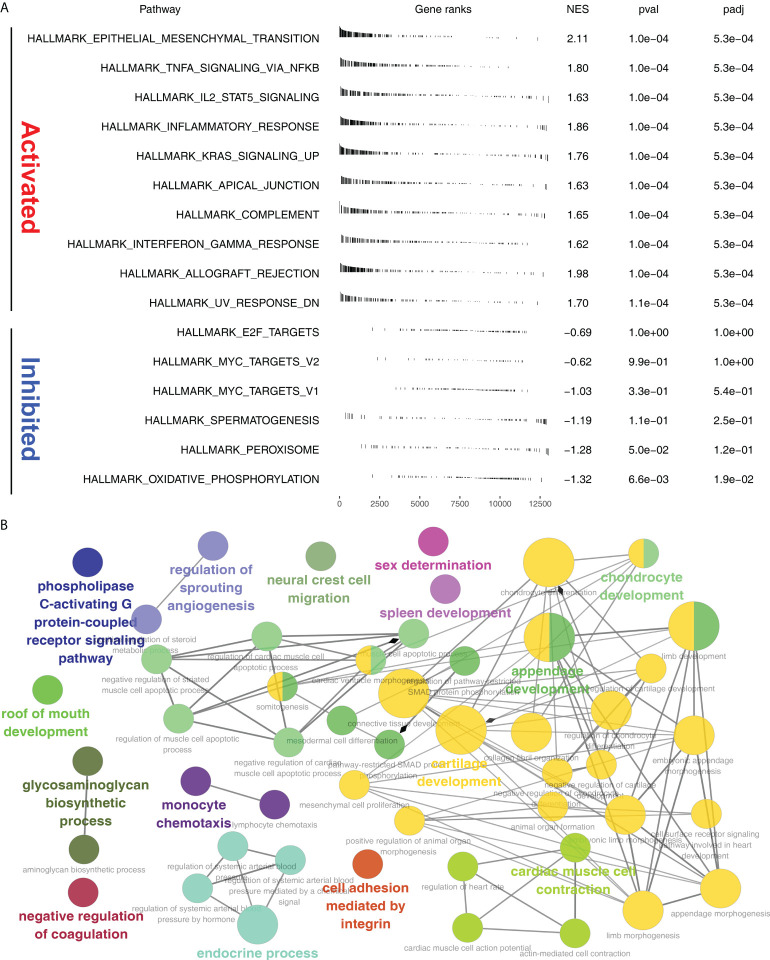
Pathway enrichment analysis. **(A)** GSEA analysis of Cluster2 based on the Hallmark gene set; **(B)** ClueGO analysis in Cytoscape software.

GSEA analysis showed that in Cluster2, the pathway of epithelial mesenchymal transition (EMT), TNFα/NF-kB signaling, IL2/STAT5 signaling, inflammatory response, KRAS signaling, apical junction, complement, interferon gamma response, allograft rejection were significantly activated (**Figure 5A**). ClueGO analysis showed that in the Cluster2, the terms of phospholipase C-activating G protein-coupled receptor signaling, regulation of sprouting angiogenesis, neural crest cell migration, sex determination, spleen development, chondrocyte development, roof of mouth development, glycosaminoglycan biosynthetic process, negative regulation of coagulation, monocyte chemotaxis, endocrine process, cell adhesion mediated by integrin, cartilage development and cardiac muscle cell contraction (**Figure 5B**). Gene ontology (GO) analysis showed that in the Cluster2, the terms of cellular ion homeostasis, negative regulation of cell differentiation, embryonic morphogenesis, metal ion homeostasis, positive regulation of cell death, positive regulation of locomotion, regulation of defense response, taxis, tissue morphogenesis were upregulated (**Supplementary Figure S4A**). Kyoto Encyclopedia of Genes and Genomes (KEGG) analysis showed that in the Cluster2, the terms of cytokine cytokine receptor interaction, focal adhesion, chemokine signaling pathway, neuroactive ligand-receptor interaction, cell adhesion molecules cams, toll-like receptor signaling pathway, ECM receptor interaction, hematopoietic cell lineage, leukocyte transendothelial migration, leishmania infection were upregulated (**Supplementary Figure S4B**).

### Genomic instability analysis

**Figure 6:**
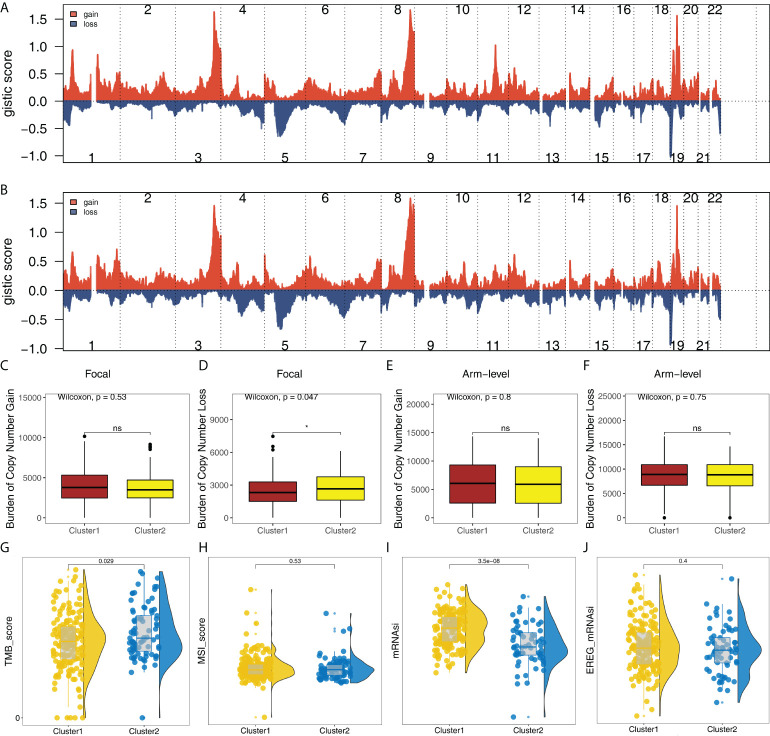
Genomic instability analysis. **(A)** The gistic score of copy number profiles of TCGA-OV in Cluster1; **(B)** The gistic score of copy number profiles of TCGA-OV in Cluster2; **(C–F)** The difference of CNV burden in focal gain, focal loss, arm-level gain and arm-level loss in Cluster1 and Cluster2 patients; **(G–J)** The difference of TMB, MSI, mRNAsi and EREG-mRNAsi in Cluster1 and Cluster2 patients. * = P < 0.05. The expanded form of ns = not significant.

In addition, the copy number profile of the OC patients in TCGA was evaluated, including the gain/loss percentage and the gistic score (**Figures 6A, B** and **Supplementary Figure S5**). CNV burden analysis showed the patients in Cluster2 might have a higher burden of copy number loss in the focal level, while no significant difference was observed in the CNV burden of other levels (**Figures 6C-F**). Moreover, we found that the patients in Cluster2 had a higher TMB_score than that in Cluster1 (**Figure 6G**). No remarkable difference was found in MSI_score (**Figure 6H**). However, we noticed that Cluster1 had a higher mRNAsi score (**Figure 6I**). No significant difference was found in EREG-mRNAsi (**Figure 6J**).

### CAFs is associated with the immunotherapy response of OC

**Figure 7:**
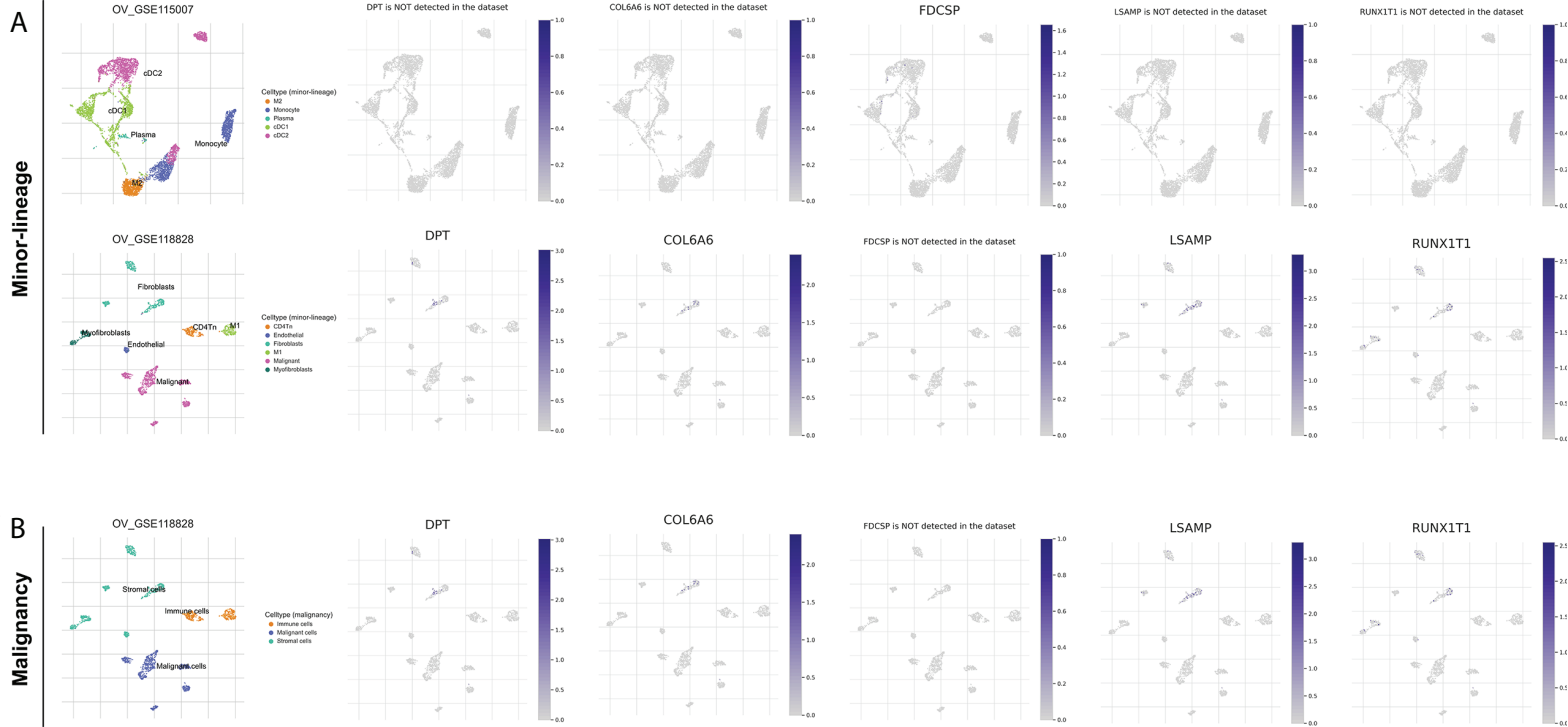
Single-cell level of DPT, RUNX1T1, LSAMP, FDCSP and COL6A6 in OC. **(A)** DPT, COL6A6, LSAMP and RUNX1T1 were mainly expressed in the fibroblasts in minor-lineage option; **(B)** DPT, COL6A6, LSAMP and RUNX1T1 were mainly expressed in the fibroblasts in and malignancy option.

**Figure 8:**
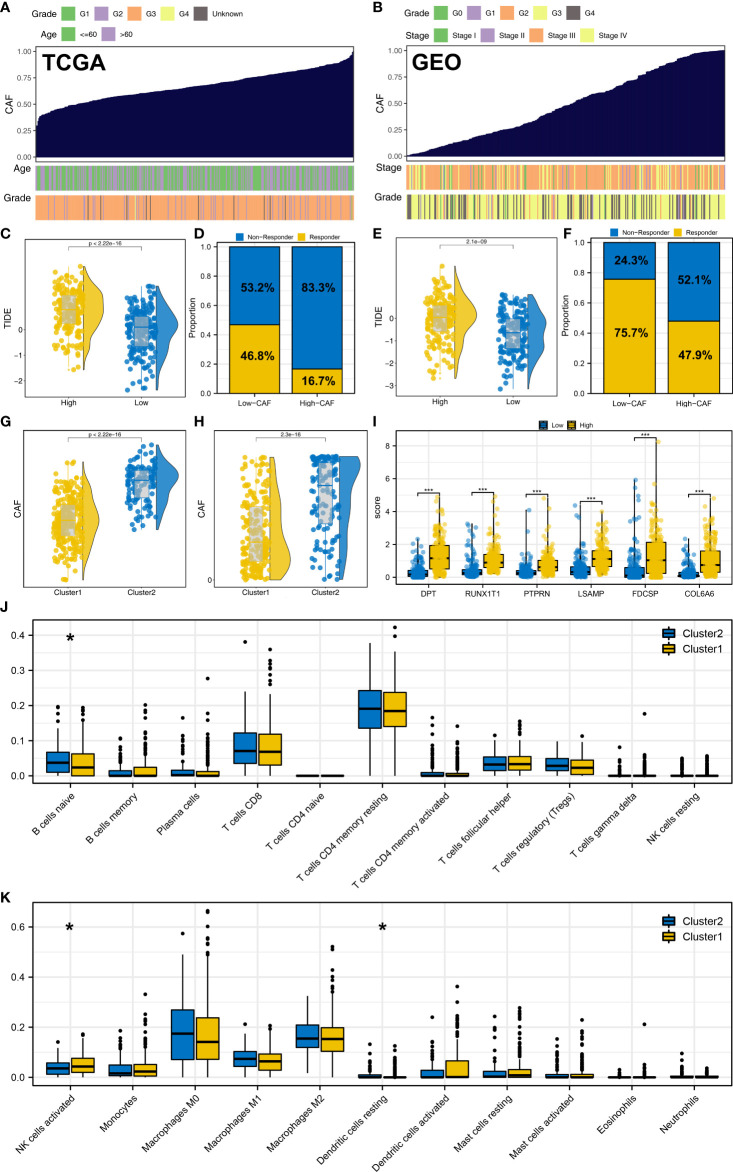
CAFs is associated with the immunotherapy response of OC. **(A, B)** ssGSEA algorithm was used to quantify the CAFs infiltration in TCGA and GSE cohorts; **(C, D)** In TCGA, patients with low CAFs infiltration had a lower TIDE score and a higher proportion of immunotherapy responders; **(E, F)** In the GEO cohort, patients with low CAFs infiltration had a lower TIDE score and a higher proportion of immunotherapy responders; **(G)** In TCGA, patients in Cluster2 had a higher CAFs infiltration; **(H)** In the GEO cohort, patients in Cluster2 also had a higher CAFs infiltration; **(I)** DPT, RUNX1T1, PTPRN, LSAMP, FDCSP and COL6A6 were upregulated in the patients with high CAFs infiltration; **(J, K)** Immune infiltration analysis of Cluster1 and Cluster2. * = P < 0.05, *** = P < 0.001.

We further explored the characteristic genes in the single-cell level of OC. The result showed that the DPT, COL6A6, LSAMP and RUNX1T1 was mainly expressed in the fibroblasts both in minor-lineage and malignancy option (**Figures 7A, B**). Therefore, we think it would be interesting to know if CAFs could affect the immunotherapy response rate in OC patients. Then, we performed ssGSEA analysis to quantify the infiltration level of CAFs in OC patients (**Figures 8A, B**). In TCGA cohort, the result showed that the patients with low CAFs infiltration might have a lower TIDE score and a higher proportion of immunotherapy responders (**Figures 8C, D**; 46.8% vs 16.7%). The same conclusion was observed in the GSE cohort (**Figures 8E, F**, 75.7% vs 47.9%). Notably, the patients in Cluster2 had a higher CAFs infiltration in both TCGA and GSE cohorts, which might partly explain the higher proportion of immunotherapy non-responders in Cluster2 (**Figures 8G, H**). Interestingly, we found all the characteristic genes DPT, RUNX1T1, PTPRN, LSAMP, FDCSP and COL6A6 were upregulated in the patients with high CAFs infiltration (**Figure 8I**). Immune infiltration analysis showed that the patients in Cluster2 might have a higher infiltration of naive B cells, activated NK cells and resting Dendritic cells (**Figures 8J, K**). Pathway enrichment analysis showed that in the patients with high CAFs infiltration, the pathways of EMT, TNF-α signaling, apical junction, IL2/STAT5 signaling, inflammatory response, allograft rejection, KRAS signaling, myogenesis, UV response, complement were activated (**Supplementary Figure S6**).

## Discussion

There is a huge public health impact associated with OC, especially since there are so many forms of OC, each with a unique biology and prognosis (20). Immunotherapy has shown promising application prospects in a variety of solid tumors (21). Also, in OC, relevant preclinical trials have been carried out with encouraging results. Therefore, prospectively exploring the internal biological mechanisms behind the patients with different response performances to immunotherapy in OC is meaningful.

Here, we performed the TIDE analysis to evaluate the immunotherapy response rate of OC patients. The machine learning algorithm LASSO logistic regression and SVM-RFE were used to identify the characteristic genes. The genes DPT, RUNX1T1, PTPRN, LSAMP, FDCSP and COL6A6 were selected for molecular typing. Our result showed that the patients in Cluster1 might have a better prognosis and might be more sensitive to immunotherapy, including PD-1 and CTLA4 therapy options. Pathway enrichment analysis showed that in Cluster2, the pathway of EMT, TNFα/NF-kB signaling, IL2/STAT5 signaling, inflammatory response, KRAS signaling, apical junction, complement, interferon-gamma response and allograft rejection were significantly activated. Also, genomic instability analysis was performed to identify the underlying genomic difference between the different Cluster patients. Single-cell analysis showed that the DPT, COL6A6, LSAMP and RUNX1T1 were mainly expressed in the fibroblasts. We then quantified the CAFs infiltration in the OC samples. The result showed that patients with low CAFs infiltration might have a lower TIDE score and a higher proportion of immunotherapy responders. Also, we found all the characteristic genes DPT, RUNX1T1, PTPRN, LSAMP, FDCSP and COL6A6 were upregulated in the patients with high CAFs infiltration. Immune infiltration analysis showed that the patients in Cluster2 might have a higher infiltration of naive B cells, activated NK cells and resting Dendritic cells.

During the past two decades, immunotherapy has evolved rapidly and revolutionized treatment options for many cancers. Recently, immune checkpoint inhibitors have been investigated for possible use in reversing immunosuppressive TME, including CTLA4 and PD-1/L1 inhibitors (22). As oncolytic viruses, cancer vaccines, and adoptive cell therapy have advanced rapidly, immunotherapy has also gained much attention in OC therapy. Currently, most types of OC immunotherapy treatment options, like CAR-T and immune checkpoint inhibitors are in clinical trials (23). Although promising approaches have been developed for OC immunotherapy, the immunosuppressive TME still needs to be overcome to improve the effectiveness of immunotherapy (24). In our study, we found that the CAFs was tightly associated with the immunotherapy response of OC patients. Previous studies have explored the role of CAFs in cancer immunotherapy. Through Single-cell analysis, Kieffer et al. identified eight CAFs clusters and they found that PD-1 and CTLA4 proteins were upregulated by cluster 0/ecm-myCAF in regulatory T lymphocytes (Tregs), which increases CAF-S1 cluster 3/TGFβ-myCAF cellular content (25). Obradovic et al. performed scRNA-seq on the cancer tissue obtained from four advanced-stage head and neck squamous cell carcinoma patients treated with the αPD-1 therapy, nivolumab (pretreatment and posttreatment). They revealed that a significant change was observed in the abundance of fibroblasts following treatment with nivolumab and they also identified different CAFs clusters, which have a potential guiding effect (26).

Six characteristic genes were identified, including DPT, RUNX1T1, PTPRN, LSAMP, FDCSP and COL6A6. In OC, Yeh et al. found that in OC, the aberrant TGFβ/SMAD4 signaling can induce epigenetic silencing of putative tumor suppressor RUNX1T1 (27). Sun et al. indicated that lncRNA EPB41L4A-AS2 hamper the development of OC by sequestering microRNA-103a and upregulating transcription factor RUNX1T1 (28). Moreover, Wang et al. indicated that FDCSP could facilitate OC metastasis by promoting cancer cell migration and invasion (29). Also, we found that DPT, COL6A6, LSAMP and RUNX1T1 were mainly disturbed in the fibroblast. Kang et al. demonstrated that COL6A6 is expressed in fibroblast and has the potential to be a target of head and neck squamous cell carcinoma (30). In osteosarcoma, Feleke et al. found that LSAMP was highly expressed in the osteoblastic osteosarcoma cells and CAFs, which have the potential to be a therapeutic target (31).

Pathway enrichment analysis showed in Cluster2, the pathway of EMT, TNFα/NF-kB signaling, IL2/STAT5 signaling was significantly activated. EMT plays an important role in promoting tumor malignant biological behavior. In OC, Wu et al. found that ST3GAL1 could contribute to migration, invasion and paclitaxel resistance in OC through EMT induced by TGF-β1 (32). Liang et al. revealed that lncRNA PTAR could promote EMT and invasion in OC by competitively binding miR-101-3p to upregulate ZEB1 expression (33). Immune infiltration analysis showed that Cluster2 had a lower infiltration level of activated NK cells. Research has demonstrated that NK cells can kill ovarian cancer cells effectively. A lower NK cells infiltration might be partly responsible for the worse prognosis of Cluster2.

Several limitations should be noted. Firstly, the population in our analysis was mainly White patients and the underlying race bias is inescapable. Asian and African large-scale sequencing data should be paid more attention in the future. Secondly, there is still no open-accessed genomic data of OC patients with immunotherapy. The response rate predicted by TIDE analysis is still affected by the bioinformatics algorithm and hard to fully reflect the real situation.

## Data availability statement

The datasets presented in this study can be found in online repositories. The names of the repository/repositories and accession number(s) can be found in the article/**Supplementary Material**.

## Author contributions

Manuscript preparation: JC. Data collection: SC. Data analysis: XD. Chart preparation: LM and YC. Research design: WB and YS. All the authors have read and approved the final draft for submission.

## Acknowledgments

The authors would like to give their sincere appreciation to the reviewers for their helpful comments on this article and research groups for the TCGA and CEO, which provided data for this collection.

## Conflict of interest

The authors declare that the research was conducted in the absence of any commercial or financial relationships that could be construed as a potential conflict of interest.

## Publisher’s note

All claims expressed in this article are solely those of the authors and do not necessarily represent those of their affiliated organizations, or those of the publisher, the editors and the reviewers. Any product that may be evaluated in this article, or claim that may be made by its manufacturer, is not guaranteed or endorsed by the publisher.

## Supplementary material

Supplementary Figure 1 The flow chart of the whole study.

Supplementary Figure 2 Molucular typing based on DPT, RUNX1T1, PTPRN, LSAMP, FDCSP and COL6A6 in TCGA database.

Supplementary Figure 3 Molucular typing based on DPT, RUNX1T1, PTPRN, LSAMP, FDCSP and COL6A6 in GSE database.

Supplementary Figure 4 GO and KEGG analysis. **(A)**: GSEA analysis of Cluster2 based on the c2.cp.kegg.v7.5.1.symbols gene set; **(B)**: GSEA analysis of Cluster2 based on the c5.go.v7.5.1.symbols gene set.

Supplementary Figure 5 GISTIC plot of Cluster1 and Cluster2. **(A)**: amp_qplot of Cluster1; **(B)**: del_qplot of Cluster2; **(C)**: amp_qplot of Cluster2; **(D)**: del_qplot of Cluster2.

Supplementary Figure 6 Pathway enrichment analysis of the CAFs.
