# Supplementary figures and images for: Exploration of the underlying biological differences and targets in ovarian cancer patients with diverse immunotherapy response
Source: Front Immunol. 2022 Sep 15;13:1007326. doi: 10.3389/fimmu.2022.1007326 (PMC9521167; doi:10.3389/fimmu.2022.1007326)

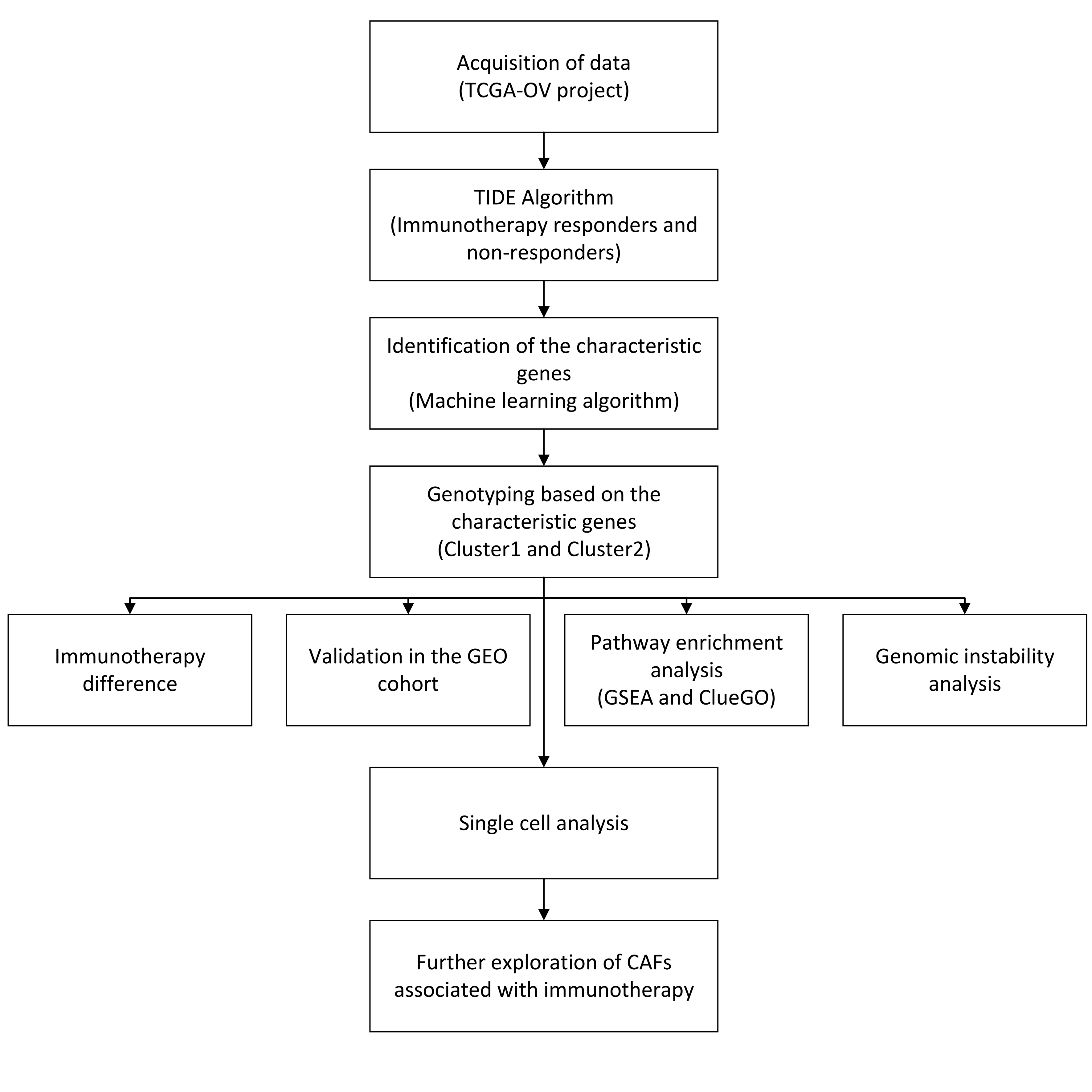

Supplement: Supplementary Figure 1 — The flow chart of the whole study. [file Image_1.tif]

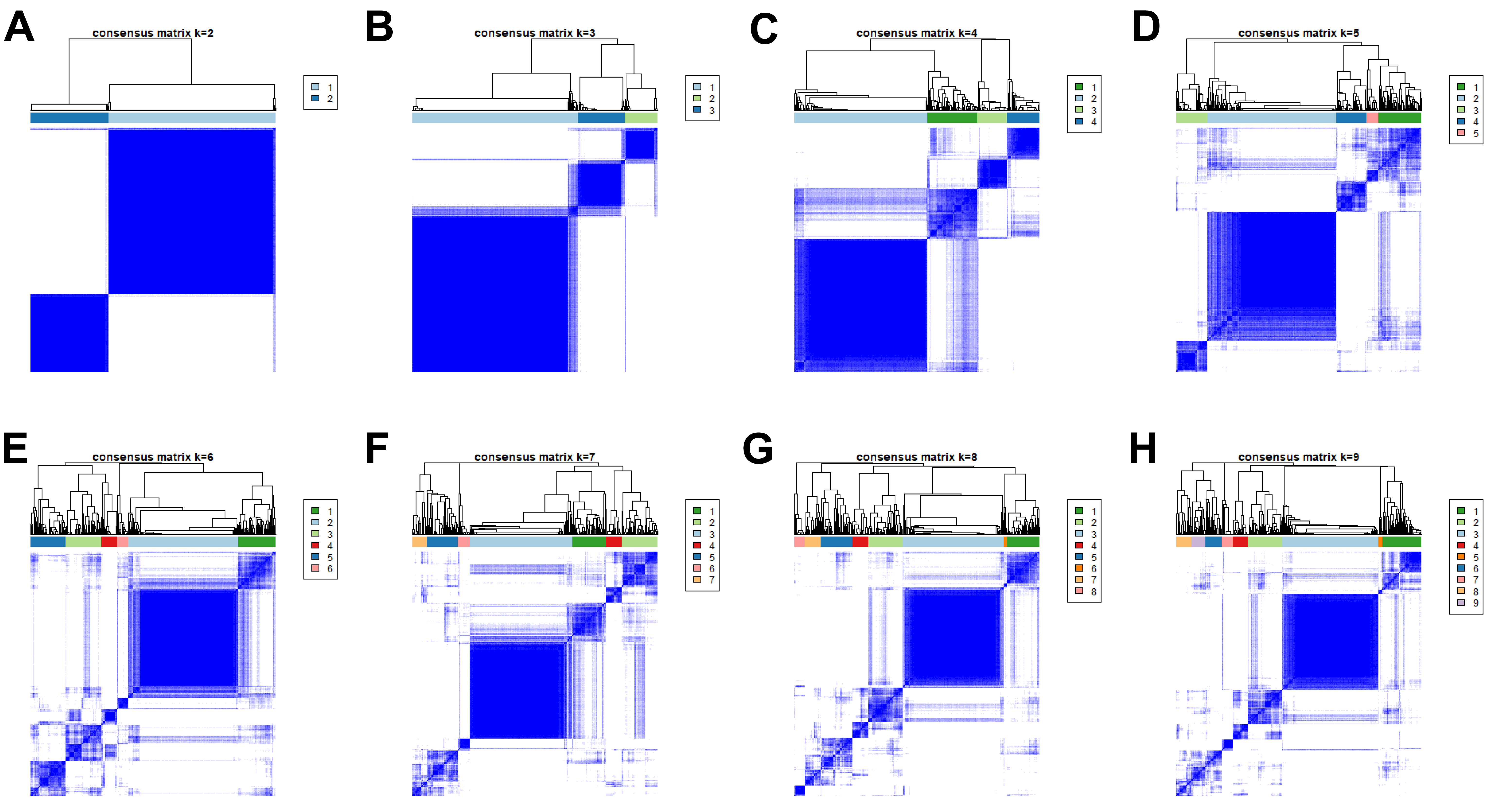

Supplement: Supplementary Figure 2 — Molucular typing based on DPT, RUNX1T1, PTPRN, LSAMP, FDCSP and COL6A6 in TCGA database. [file Image_2.tif]

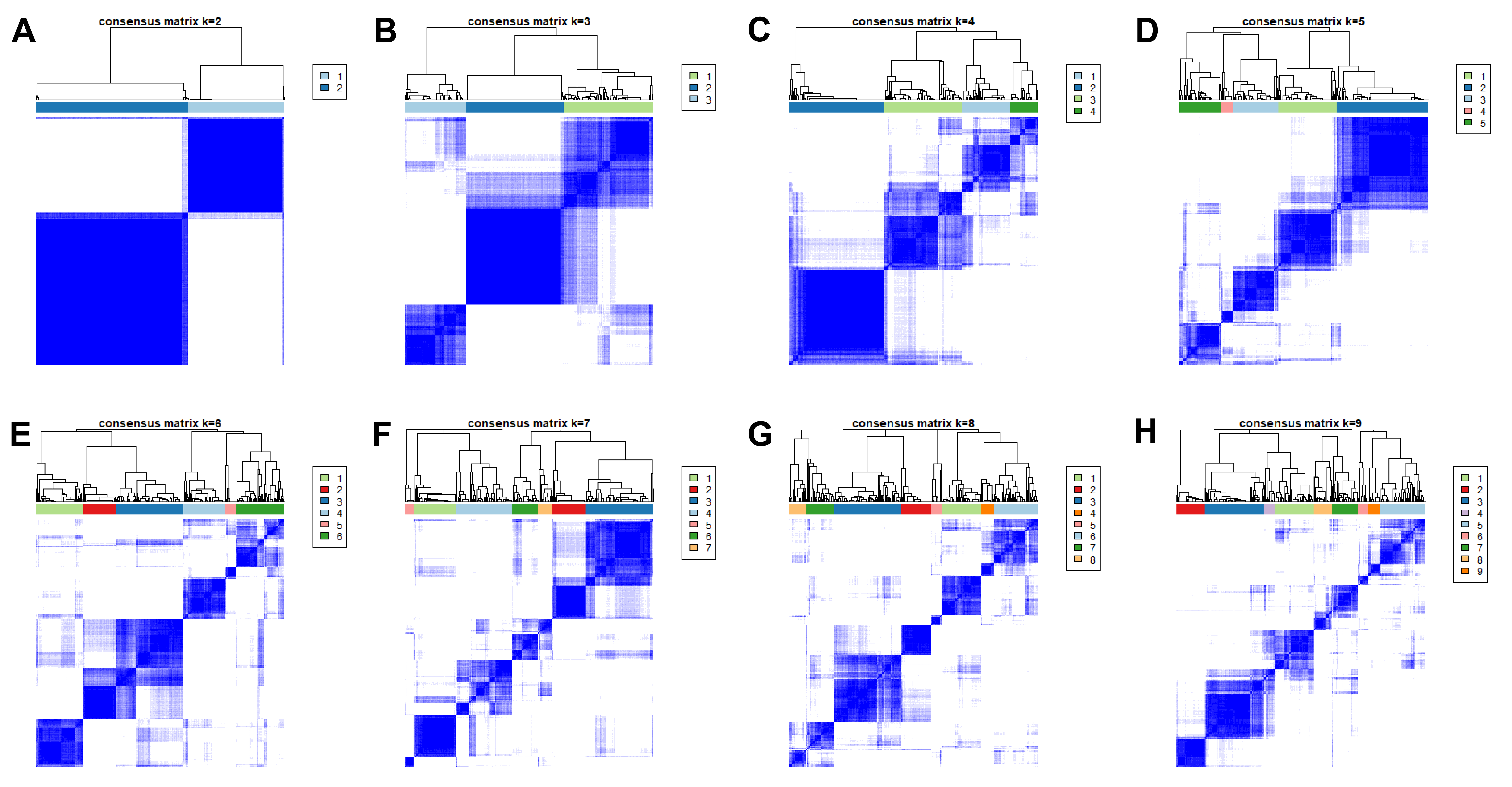

Supplement: Supplementary Figure 3 — Molucular typing based on DPT, RUNX1T1, PTPRN, LSAMP, FDCSP and COL6A6 in GSE database. [file Image_3.tif]

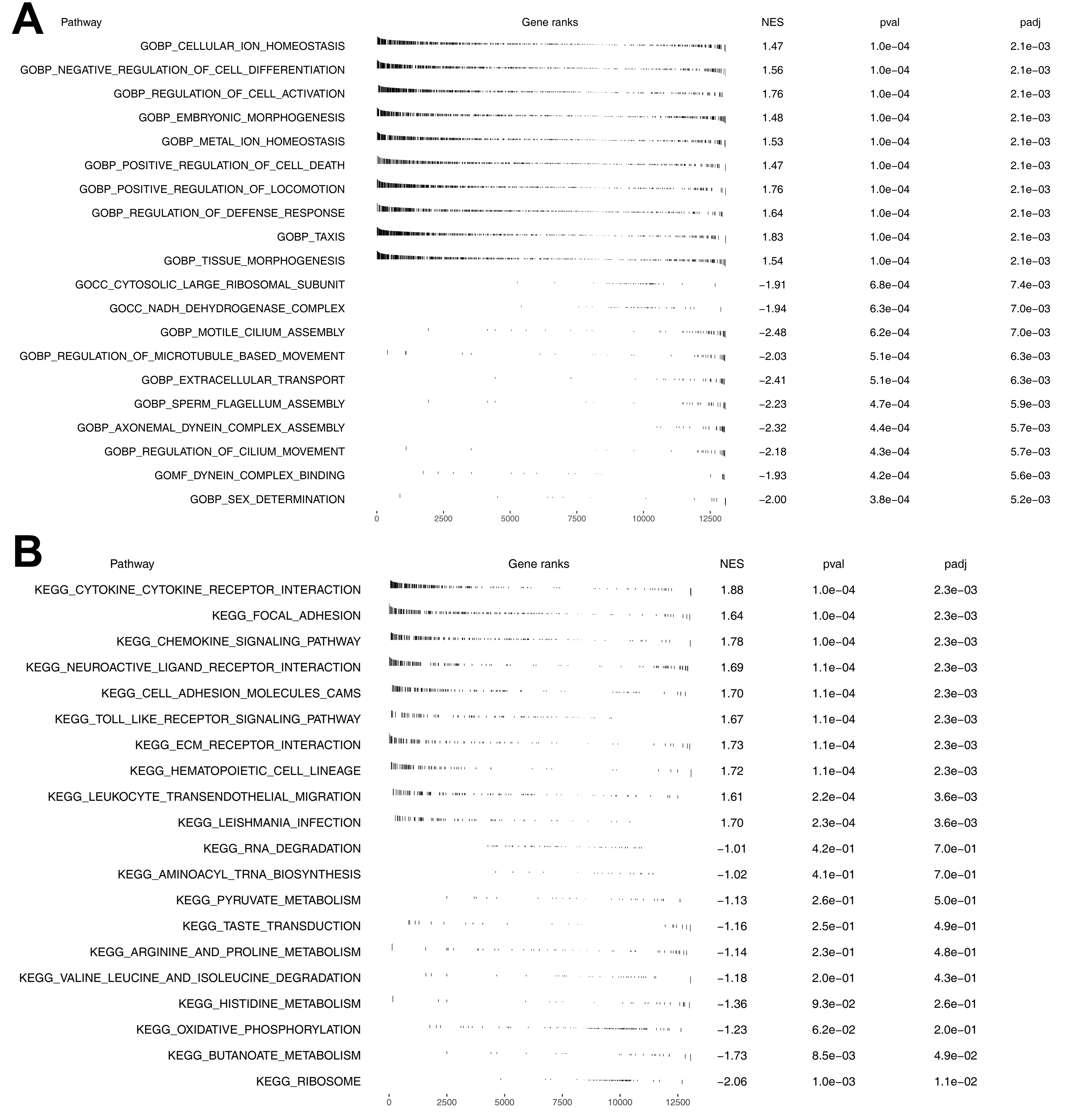

Supplement: Supplementary Figure 4 — GO and KEGG analysis. (A): GSEA analysis of Cluster2 based on the c2.cp.kegg.v7.5.1.symbols gene set; (B): GSEA analysis of Cluster2 based on the c5.go.v7.5.1.symbols gene set. [file Image_4.tif]

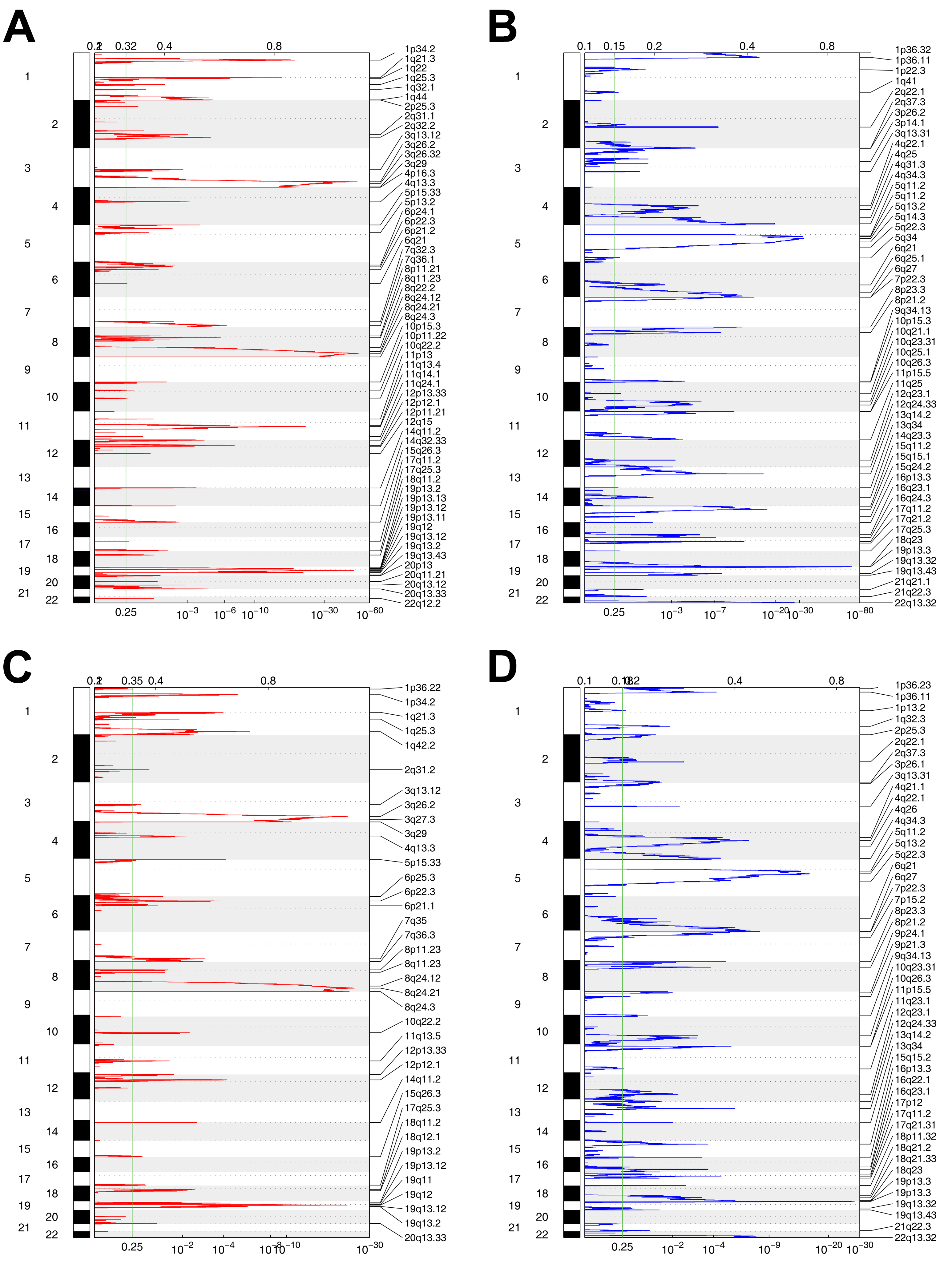

Supplement: Supplementary Figure 5 — GISTIC plot of Cluster1 and Cluster2. (A): amp_qplot of Cluster1; (B): del_qplot of Cluster2; (C): amp_qplot of Cluster2; (D): del_qplot of Cluster2. [file Image_5.tif]

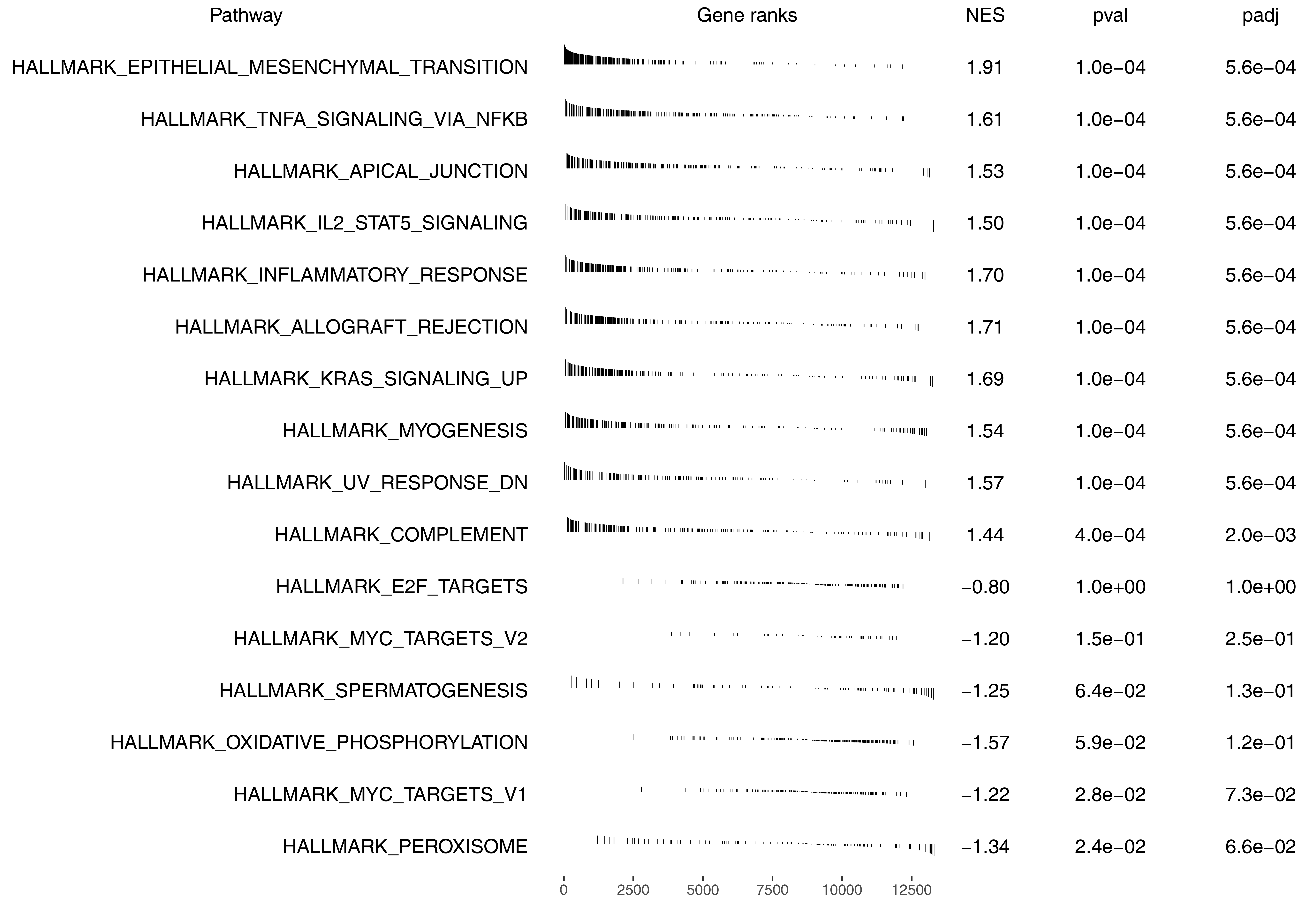

Supplement: Supplementary Figure 6 — Pathway enrichment analysis of the CAFs. [file Image_6.tif]
