# Supplementary material for: Exploration of the underlying biological differences and targets in ovarian cancer patients with diverse immunotherapy response
Source: Front Immunol. 2022 Sep 15;13:1007326. doi: 10.3389/fimmu.2022.1007326 (PMC9521167; doi:10.3389/fimmu.2022.1007326)
Supplement: Supplementary file 7 [file Table_1.docx]

**Table S1. The reference genes for CAFs quantification**

| **Algorithm** | **Reference gene symbol** |
| --- | --- |
| ssGSEA | ACTA2, FAP, PDGFRB, CAV1, PDPN, PDGFRA, ZEB1, FOXF1, SPARC, MMP2, FN1 |
